# Epidemiology of Acute Pancreatitis in Hospitalized Children in the United States from 2000–2009

**DOI:** 10.1371/journal.pone.0095552

**Published:** 2014-05-07

**Authors:** Chaitanya Pant, Abhishek Deshpande, Mojtaba Olyaee, Michael P. Anderson, Anas Bitar, Marilyn I. Steele, Pat F. Bass, Thomas J. Sferra

**Affiliations:** 1 Department of Medicine, Kansas University Medical Center, Kansas City, Kansas, United States of America; 2 Medicine Institute Center for Value Based Care, Cleveland Clinic, Cleveland, Ohio, United States of America; 3 Department of Biostatistics and Epidemiology, University of Oklahoma Health Sciences Center, Oklahoma City, Oklahoma, United States of America; 4 Department of Pediatrics, University of Oklahoma Health Sciences Center, Oklahoma City, Oklahoma, United States of America; 5 Departments of Medicine and Pediatrics, Louisiana Health Sciences Center-Shreveport, Shreveport, Louisiana, United States of America; 6 Department of Pediatrics, Case Western Reserve University School of Medicine, UH Rainbow Babies & Children’s Hospital, Cleveland, Ohio, United States of America; University of Szeged, Hungary

## Abstract

**Background:**

Single-center studies suggest an increasing incidence of acute pancreatitis (AP) in children. Our specific aims were to (i) estimate the recent secular trends, (ii) assess the disease burden, and (iii) define the demographics and comorbid conditions of AP in hospitalized children within the United States.

**Methods:**

We used the Healthcare Cost and Utilization Project Kids’ Inpatient Database, Agency for Healthcare Research and Quality for the years 2000 to 2009. Extracted data were weighted to generate national-level estimates. We used the Cochrane-Armitage test to analyze trends; cohort-matching to evaluate the association of AP and in-hospital mortality, length of stay, and charges; and multivariable logistic regression to test the association of AP and demographics and comorbid conditions.

**Results:**

We identified 55,012 cases of AP in hospitalized children (1–20 years of age). The incidence of AP increased from 23.1 to 34.9 (cases per 10,000 hospitalizations per year; P<0.001) and for all-diagnoses 38.7 to 61.1 (P<0.001). There was an increasing trend in the incidence of both primary and all-diagnoses of AP (P<0.001). In-hospital mortality decreased (13.1 to 7.6 per 1,000 cases, P<0.001), median length of stay decreased (5 to 4 days, P<0.001), and median charges increased ($14,956 to $22,663, P<0.001). Children with AP compared to those without the disease had lower in-hospital mortality (adjusted odds ratio, aOR 0.86, 95% CI, 0.78–0.95), longer lengths of stay (aOR 2.42, 95% CI, 2.40–2.46), and higher charges (aOR 1.62, 95% CI, 1.59–1.65). AP was more likely to occur in children older than 5 years of age (aORs 2.81 to 5.25 for each 5-year age interval). Hepatobiliary disease was the comorbid condition with the greatest association with AP.

**Conclusions:**

These results demonstrate a rising incidence of AP in hospitalized children. Despite improvements in mortality and length of stay, hospitalized children with AP have significant morbidity.

## Introduction

Acute pancreatitis (AP) is defined as reversible inflammation of the pancreatic parenchyma and is characterized by the presence of interstitial edema, acute inflammatory cell infiltrate, and varying degrees of cellular apoptosis, necrosis and hemorrhage [1]. The INSPPIRE (International Study Group of Pediatric Pancreatitis: In Search for a Cure) consortium recently operationally defined the diagnosis of AP as requiring two of the following: (i) abdominal pain compatible with AP, (ii) serum amylase or lipase levels greater or equal to three times the upper limits of normal, and (iii) imaging findings consistent with AP [2]. In children, the most frequently identified etiologies of AP are biliary tract disease, medication adverse effect, systemic disease, and trauma (reviewed in Bai *et al*) [3]. In recent years, several single-center studies from the United States and the rest of the world have reported an increase in the number of cases of AP in children [4]–[9]. The identified basis for the increase in pediatric AP cases differ amongst the reporting centers and include increases in: (i) testing of children for AP [5], (ii) specific etiologies of AP [6], and (iii) referral of children with AP to a tertiary care center [7].

Two studies specifically examined the population-based incidence (not only the absolute number) of cases of AP in children. Nydegger and colleagues retrospectively evaluated cases at a large pediatric referral and trauma center (The Royal Children’s Hospital) in Melbourne, Australia from 1993 through 2002 [6]. They found an increase in the annual incidence of pancreatitis from 24.6±2.3 cases per year per 100,000 children for the initial 5-year period to 31.2±6.0 for the last 5-year period of the study. This change in incidence of AP was determined to be due to primarily an increase in the number of cases of AP associated with systemic diseases and those with an unidentified (idiopathic) etiology. Morinville and colleagues reviewed cases of AP admitted to the Children’s Hospital of Pittsburgh from 1993 to 2004 [5]. During this period of time, the incidence of first time admission for AP increased over 5-fold from 2.4 to 13.2 per 100,000 children. The authors determined that an increase in the frequency of testing serum amylase and lipase levels accounted for nearly all of the observed increase in AP admissions.

These previous studies demonstrate that the absolute number of children diagnosed with AP at several individual institutions has risen. However, the results of studies involving single tertiary care centers cannot be applied necessarily to larger populations and it is unclear whether the increase in disease has continued in more recent years. Also, better characterization of AP in children is needed [2] especially in light of the possible increase in the number of children hospitalized with this disease. Therefore, the goal of this study was to examine the recent epidemiology of acute pancreatitis in children using multi-institutional data. We utilized a United States pediatric inpatient database to estimate the recent secular trends of pediatric AP, assess the disease burden of AP in hospitalized children, and define the demographics and comorbid conditions associated with this disease.

## Methods

### Data Source

We used the Healthcare Cost and Utilization Project Kids’ Inpatient Database (HCUP-KID) sponsored by the Agency for Healthcare Research and Quality to obtain the data for this study. The HCUP-KID contains data from community hospitals, as defined by the American Health Association, within the United States. Community hospitals are nonfederal, short-term general, and special hospitals (e.g. children’s hospitals, obstetrics and gynecology, orthopedic) accessible by the general public. These hospitals can be academic medical centers or other teaching hospitals. Individual hospitalizations (i.e. discharge level, not patient level information is collected) of patients who are 20 years of age or less at the time of admission are de-identified and maintained in the HCUP-KID as unique entries. The structure of the HCUP-KID (e.g. discharge level entries, no individual patient identifiers) precludes longitudinal tracking of individual patients. Thus, patients readmitted to a hospital or transferred from one to another cannot be identified. Each discharge entry includes 1 primary discharge diagnosis and 1–24 secondary diagnoses (based on the International Classification of Diseases, Ninth Revision, and Clinical Modification, ICD-9-CM, diagnosis codes). The HCUP-KID provides an individual-level population weight to generate national level estimates of total cases. All data in this manuscript are presented as national level estimates. For each year included in this study (2000, 2003, 2006, and 2009) the HCUP-KID contains between 7,291,032 and 7,370,203 weighted total pediatric cases reported from between 2,784 and 4,121 hospitals.

### Variable Definition

The predictor variable in this study was the presence of a diagnosis of AP. We used the ICD-9-CM diagnostic code for AP (577.0) to identify cases with this diagnosis. This is the sole code for AP and it has be used previously to identify AP cases in children [5]. We extracted all entries with a primary or secondary discharge diagnosis of AP. We distinguished between primary and all-diagnoses (primary and secondary diagnoses combined) for the purpose of calculating the incidence and trends of AP. For all other analyses, we used all-diagnoses of AP. We excluded children less than 1 year of age from our analysis. Newborn infants as part of routine hospital deliveries comprise a significant proportion of entries in the HCUP-KID (e.g. uncomplicated births accounted for approximately 38% of all hospital discharges in 2009). The addition of this age group would introduce a large number of healthy infants to the data set. This would skew the analysis of demographic and outcome parameters. In a preliminary analysis we identified less than 300 cases of AP in patients less than 1 year of age for all the years under study.

For each case, we extracted the following demographic details: year of diagnosis, age, gender, hospital setting and teaching status, U.S. geographic region, household income, and insurance status. Household income was classified according to income quartiles (1^st^–4^th^) per HCUP documentation based on patient residence. Race is incompletely reported within the KID (i.e. four states do not report race) and, thus, was not included in this study. To evaluate disease burden, we used the outcome variables of in-hospital mortality, length of hospital stay (LOS), and hospitalization charges. Charges were adjusted to 2009 dollars using the consumer price index.

### Evaluation of Associated Comorbid Conditions

ICD-9 diagnostic codes for comorbid diagnoses in patients with AP were placed into clinically meaningful categories based on a modified version of the clinical categories described by Rassekh *et al* [10]. These clinical categories were then ranked in order of frequency. For our analysis, we included categories that occurred in 2% or greater of the extracted cases of AP or if a category had been recognized as strongly associated with AP.

### Statistical Analysis

Statistical analyses were performed using SAS version 9.2 (SAS institute, Cary, North Carolina). Data were tested for normality using the Kilgomorov-Smirnoff test. Categorical data are reported as frequencies and percentages. Normally distributed continuous data are reported as mean values with standard errors; data not normally distributed are reported as median values with interquartile range (IQR). The Kruskal-Wallis and ANOVA test were used for comparing differences between non-normal and normal continuous variables respectively. The Chi-square test was used for comparing differences between categorical variables. For trend analysis we utilized the Cochrane-Armitage test. The threshold for significance for these analyses was P<0.05.

To account for confounders that were likely to be associated with both AP and the outcomes under study (in-hospital mortality, LOS, and hospitalization charges), cohort-matching using high-dimensional propensity scores was performed. High-dimensional propensity scores were generated by regression analysis of patients with AP based on their demographics and the identified comorbid conditions as described previously in this section under the heading “Evaluation of Associated Comorbid Conditions.” Patients with AP were matched by high-dimensional propensity score, using a greedy matching algorithm, to patients who did not have AP with a 1∶5 matching ratio [11]. The Chi-square test was used to calculate odds ratios (ORs) and 95% confidence intervals (CIs). CIs are reported to identify the strength and significance of AP and other covariates on the likelihood of the outcome variables.

To assess demographics and comorbid conditions associated with AP among all pediatric inpatients, univariate and multivariable logistic regression analyses were performed with the presence or absence of AP as the dichotomous outcome variable. Covariates that were tested for association with AP in this manner included demographics and the identified comorbid conditions as previously described in this section. We tested all between-variable estimated correlation coefficients and determined that multicollinearity was not a problem. ORs, adjusted odds ratios (aORs), and 95% confidence intervals (CIs) are reported to identify the strength and significance of AP and other covariates on the likelihood of an association.

## Results

For the period of our study (2000–2009), there were a total of 11,022,792 discharges for children over 1 year of age of which 55,012 had a diagnosis of AP. Demographic characteristics of both the AP cases and all other cases in the database without AP are listed in Table 1. There were significant statistical differences between the two groups in the majority of the demographics studied including age, sex, hospital setting, region of care, household income, and insurance status (P<0.001). Age is the most likely clinically important of these significant differences as it markedly differed between the two groups. Notably, greater than 80% of cases with AP as compared to approximately 64% without AP were over 11 years of age (P<0.001). This difference in age distribution, also, was observed when we evaluated the incidence of AP by study year (Table 2).

**Table 1:** Characteristics of combined cases for the years 2000, 2003, 2006, and 2009 with and without a discharge diagnosis of AP.

Variable^a^	Acute Pancreatitis	Without Acute Pancreatitis
**Number (N)**	55,012	10,967,780
**Age (median, IQR, years)**	17 (6)	15 (13)
**Age group (%)**		
1–5 years	6.7	23.3
6–10	10.1	12.5
11–15	20.4	16.0
16–20	62.8	48.2
**Sex (%)**		
Female	63.1	60.4
Male	36.3	38.9
**Hospital Setting (%)**		
Rural	11.1	12.9
Urban	85.1	84.1
**Teaching Status (%)** ^b^		
Teaching	55.1^N.S.^	55.3^N.S.^
Non-teaching	41.2^N.S.^	41.7^N.S.^
**Geographic region (%)**		
Northeast	15.4	17.9
Midwest	21.0	22.1
South	37.7	38.6
West	26.0	21.3
**Household Income (%)** ^c^		
1^st^ quartile	27.0^N.S.^	26.6^N.S.^
2^nd^	26.8^N.S.^	27.2^N.S.^
3^rd^	23.4^N.S.^	23.4^N.S.^
4^th^	21.0	20.2
**Insurance status (%)**		
Private	44.9	43.2
Public	40.7	46.6
None	14.2	10.0

a For each demographic variable there are significant differences (P<0.001) between patients with and without AP, except for those numbers indicated by a superscript N.S.

b,c For those demographic categories with non-significant variables overall P values were calculated: ^b^overall P = 0.414, ^c^overall P<0.001.

**Table 2:** Incidence^a^ of cases with a discharge diagnosis of AP.

	Age group (years)^b^			
	1–5	6–10	11–15	16–20
**Year**	**Principal Diagnosis**			
2000	5.3	21.6	31.1	28.9
2003	5.7	22.7	35.0	38.2
2006	8.4	25.8	44.1	40.7
2009	8.2	30.8	48.3	44.5
	**All-diagnoses**			
2000	12.4	34.1	50.6	47.8
2003	12.3	38.2	56.8	63.0
2006	16.4	41.0	69.1	68.8
2009	16.8	48.9	77.6	80.0

a Incidence is calculated as the number of cases per 10,000 discharges during each specified year.

b For each year, the incidence rates are significantly different between each age group, analyses based upon unrounded values (P<0.001). For each age group there is a significant increasing trend in the incidence of AP for both principal and all diagnoses groups (P<0.001).

**Figure 1:**
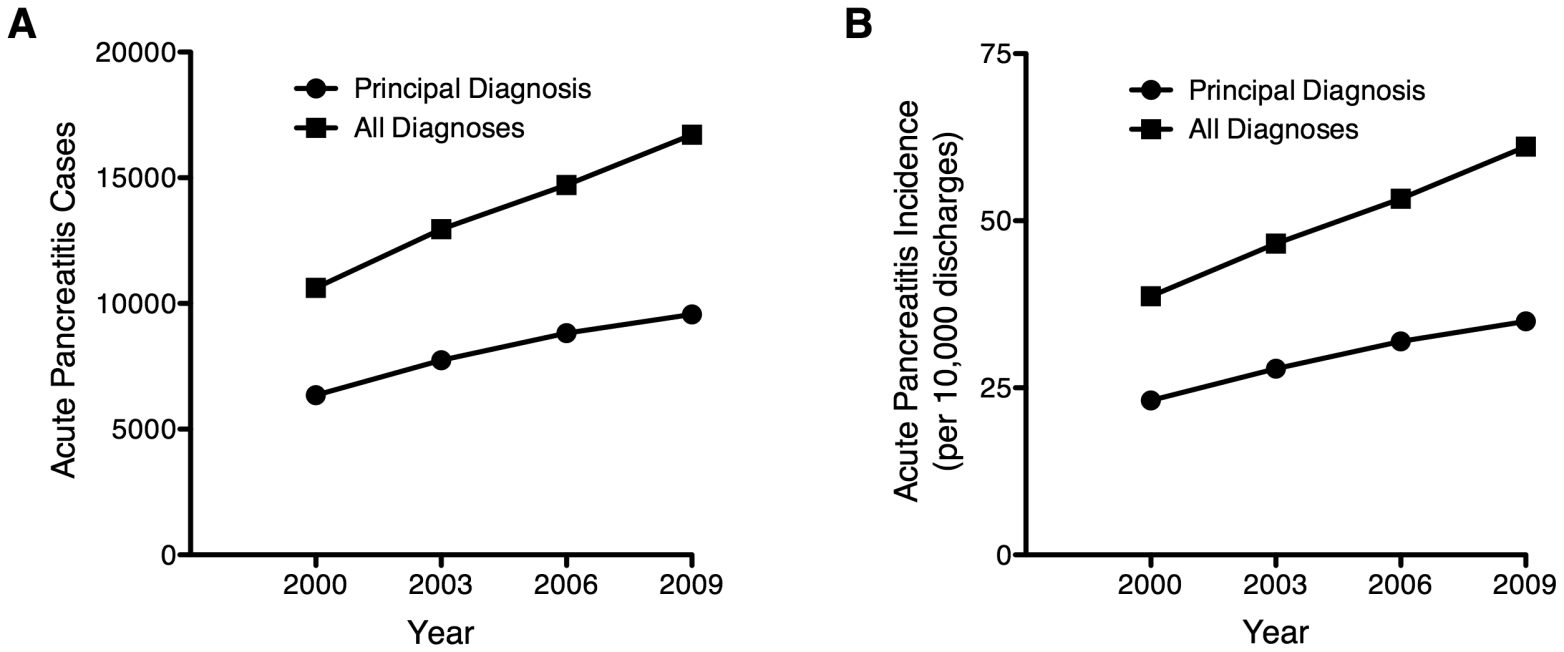
Estimated number of weighted cases (panel A) and incidence (panel B) of acute pancreatitis in hospitalized children based upon discharge diagnoses.

**Figure 2:**
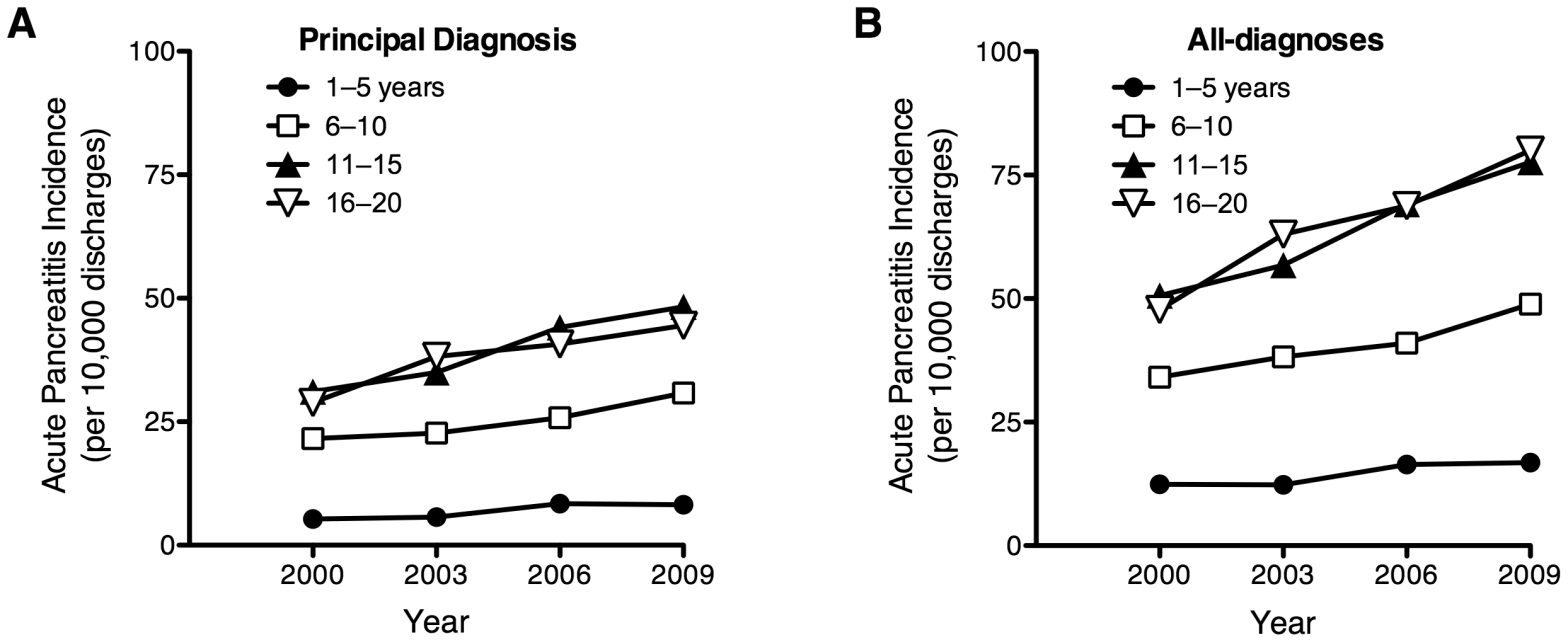
Age-based incidence of acute pancreatitis as the principal diagnosis (panel A) and all-diagnoses (panel B) in hospitalized children based upon discharge diagnoses.

During the 10-year period of study, there was a large and significant increase in the number of AP cases (Figure 1). When only cases with a primary diagnosis of AP were considered, the number increased from 6,350 in 2000 to 9,561 in 2009; this represents a 51% increase in the incidence of AP (23.1 to 34.9 cases per 10,000 discharges per year, P<0.001). When all-diagnoses of AP were considered, the number of cases increased from 10,622 in 2000 to 16,719 in 2009 (58% increase, 38.7 to 61.1 cases per 10,000 discharges per year, P<0.001). We next analyzed the trends in the incidences of AP. Overall there was a significant increase in the incidence of both primary and all-diagnoses of AP from 2000–2009 (P<0.001). Furthermore, this significant trend in the incidence of both primary and all-diagnoses of AP occurred in each of the age categories evaluated in this study (P<0.001; Table 2 and Figure 2).

We used three different outcome variables as measures of disease burden: (i) length of hospital stay, (ii) hospital charges, and (iii) mortality (Table 3). An overall comparison of the AP and non-AP cases revealed that children with AP in 2009 had a longer length of stay, higher charges, and greater mortality (P<0.001 for each). The median length of hospital stay decreased significantly for AP patients from a median of 5 to 4 days between 2000 and 2009 (P<0.001). In patients without AP, the median length of stay remained unchanged at 2 days. There was a significant decrease in mortality in children with AP from 13.1 to 7.6 (per 1,000 cases, P<0.001). There also was a significant decrease in mortality in children without AP from 3.4 to 2.7 (per 1,000 cases, P<0.001); this was less compared to patients with AP (21% decrease versus 42% decrease; P<0.001). Finally, there was a significant increase in total hospitalization charges for patients with AP from a median of $14,956 to $22,663 (P<0.001). Comparatively, in patients without AP, the total hospitalization charges increased from a median of $6,320 to $11,364 (P<0.001). In this instance, the increase was greater in patients without AP than in patients with AP (80% increase versus 52% increase; P<0.001).

**Table 3:** Disease burden outcome measures from 2000–2009 for cases with and without the discharge diagnosis of AP.

	LOS^a^^,^ b		Charges^a^^,^ b		Mortality^a^^,^ b ^,^ c	
	(median, IQR, days)		(median, IQR, $)			
Year	AP	non-AP	AP	non-AP	AP	non-AP
2000	5	2	14,956	6,320	13.1	3.4
	(3–8)	(1–4)	(7,521–33,032)	(3,766–11,575)		
2003	4	2	17,610	8,068	9.4	3.1
	(3–8)	(1–4)	(9,174–37,017)	(4,786–14,920)		
2006	4	2	19,989	9,527	8.3	2.9
	(3–7)	(2–4)	(10,540–40,634)	(5,613–17,676)		
2009	4	2	22,663	11,364	7.6	2.7
	(2–7)	(2–4)	(11,943–45,728)	(6,622–21,224)		

a P<0.001 for AP during 2000 versus 2009 for LOS, charges, and mortality.

b P<0.001 for AP versus non-AP for 2009 for LOS, charges, and mortality.

c Mortality expressed as number per 1,000 discharges (cases).

The association of AP with each of the above outcome variables was further analyzed using a cohort of patients with and without AP for all years under study in a 1∶5 ratio matched for demographic and comorbid conditions (Table 4). A total of 51,252 children with AP were correspondingly matched to children without AP; none of the matched variables were significantly different between the two groups at the P<0.05 level. In this analysis, AP was independently associated with an increase in the length of hospital stay (comparison to median value, aOR 2.42, 95% CI, 2.40, 2.46) and higher hospitalization charges (comparison to median value, aOR 1.62, 95% CI, 1.59, 1.65). AP was associated with lower mortality (aOR 0.86, 95% CI, 0.78, 0.95).

**Table 4:** Association between severity outcome measures from 2000–2009 for cases with and without the discharge diagnosis of AP.

Variable	aAdjusted Odds Ratio (95% Confidence Interval)	
	non-AP (reference)	AP
LOS (median)	1	2.42 (2.40, 2.46)
Charges^b^ (median)	1	1.62 (1.59, 1.65)
Mortality (%)	1	0.86 (0.78, 0.95)

a Matching was based on high, dimensional propensity scores. LOS and charges are modeled as dichotomous with a median cut point. Mortality is modeled as dichotomous outcome.

b Adjusted for inflation to 2009 dollars using the consumer price index.

To explore the association of demographics (Table 5) and comorbid conditions (other diagnoses, Table 6) in hospitalized children with AP (all-diagnoses) we used logistic regression analyses. AP was more likely to occur in children older than 5 years of age with aORs between 2.81 and 5.25 for each 5-year age interval evaluated. AP occurred slightly more frequently in females (aOR 1.25; 95% CI, 1.22, 1.27), urban hospitals (aOR, 1.08; 95% CI, 1.05, 1.12), the Western region of the United States (aOR, 1.37; 95% CI, 1.33, 1.41), and uninsured patients (aOR, 1.17; 95% CI, 1.14, 1.20). The comorbid diagnosis that was most highly associated with AP was liver and biliary disease (aOR 25.37; 95% CI, 24.85, 25.91).

**Table 5:** Univariate and multivariable association of AP (all-diagnoses) with demographic parameters.

Demographic Parameters	OR (95% CI)^a^	aOR (95% CI)^b^
**Year**		
2000	1	1
2003	1.21 (1.18, 1.24)	1.11 (1.08, 1.15)
2006	1.38 (1.35, 1.42)	1.17 (1.14, 1.20)
2009	1.58 (1.55, 1.62)	1.20 (1.16, 1.23)
**Age (years)**		
0–5	1	1
6–10	2.81 (2.69, 2.93)	2.81 (2.69, 2.94)
11–15	4.40 (4.23, 4.56)	3.91 (3.76, 4.07)
16–20	4.51 (4.36, 4.66)	5.25 (5.06, 5.46)
**Gender**		
Male	1	1
Female	1.12 (1.10, 1.14)	1.25 (1.22, 1.27)
**Hospital Setting**		
Rural	1	1
Urban	1.17 (1.14, 1.20)	1.08 (1.05, 1.12)
**Teaching status**		
Non-teaching	1	1
Teaching	1.00 (0.98, 1.00)	0.98 (0.96, 1.00)
**Geographic region**		
Northwest	1	1
Midwest	1.10 (1.07, 1.14)	1.08 (1.05, 1.11)
South	1.14 (1.11, 1.16)	1.13 (1.10, 1.16)
West	1.42 (1.38, 1.46)	1.37 (1.33, 1.41)
**Household income**		
1^st^	1	1
2^nd^	1.00 (0.97, 1.02)	1.01 (0.99, 1.04)
3^rd^	1.01 (0.99, 1.04)	0.98 (0.95, 1.01)
4^th^	1.05 (1.03, 1.08)	1.02 (0.99, 1.05)
**Insurance**		
Private	1	1
Public	0.84 (0.83, 0.86)	1.10(1.07, 1.12)
None	1.37 (1.34, 1.41)	1.17 (1.14, 1.20)

a Univariate models were constructed using regression analysis of cases with acute pancreatitis based on individual covariates only.

b Multivariable models were constructed using regression analysis of cases with AP based on interaction with both comorbid conditions and demographic parameters (age, sex, race, hospital region, median household income, payer type, and year of discharge).

**Table 6:** Univariate and multivariable association of AP (all-diagnoses) with comorbid conditions.

Comorbid disease	OR (95% CI)^a^	aOR (95% CI)^b^
Liver, biliary disease	44.44 (43.64, 45.25)	25.37 (24.85, 25.91)
Inflammatory bowel disease	4.38 (4.14, 4.63)	1.99 (1.87, 2.12)
Diabetes mellitus	3.38 (3.28, 3.49)	1.90 (1.83, 1.97)
Nutritional, fluid, electrolyte disorders	2.71 (2.67, 2.76)	1.89 (1.85, 1.93)
Thoracic, abdominal, pelvic trauma	2.01 (1.90, 2.12)	1.86 (1.75, 1.97)
Sepsis, Bacteremia	3.61 (3.46, 3.75)	1.79 (1.70, 1.88)
HIV infection	2.82 (2.48, 3.22)	1.73 (1.49, 2.01)
Renal disease	3.60 (3.46, 3.73)	1.56 (1.49, 1.63)
Other gastrointestinal disease	2.23 (2.19, 2.27)	1.41 (1.38, 1.44)
Rheumatologic disease	2.57 (2.40, 2.76)	1.25 (1.16, 1.35)
Hematopoeitic stem cell transplantation	2.67 (2.37, 3.01)	1.21 (1.05, 1.40)
Cardiovascular disease	2.59 (2.52, 2.76)	1.17 (1.13, 1.21)
Anemia	1.72 (1.68, 1.77)	1.06 (1.03, 1.09)
Coagulopathies	3.11 (2.99, 3.23)	1.06 (1.01, 1.11)
Substance abuse	1.65 (1.60, 1.69)	1.02 (0.99, 1.05)
Other infectious diseases	1.13 (1.08, 1.18)	0.97 (0.92, 1.01)
Endocrine disorders	1.94 (1.85, 2.04)	0.94 (0.89, 0.99)
Respiratory diseases	0.95 (0.93, 0.98)	0.91 (0.89, 0.94)
Cancer	1.05 (1.00, 1.09)	0.88 (0.84, 0.93)
Pneumonia	0.56 (0.54, 0.59)	0.71 (0.68, 0.75)
Neurological disorders	0.85 (0.83, 0.87)	0.70 (0.68, 0.72)
Genitourinary infections	0.81 (0.76, 0.86)	0.55 (0.52, 0.59)
Cystic fibrosis	1.68 (1.53, 1.85)	0.51 (0.47, 0.57)
Solid organ transplantation	1.77 (1.62, 1.94)	0.49 (0.44, 0.54)
Pregnancy complications	0.20 (0.20, 0.21)	0.18 (0.18, 0.19)

a Univariate models were constructed using regression analysis of cases with AP based on individual covariates only.

b Multivariable models were constructed using regression analysis of cases with AP based on interaction with both demographic parameters and comorbid conditions (age, sex, race, hospital region, median household income, payer type, and year of discharge).

## Discussion

To date, this is the largest investigation in terms of number of cases of AP in hospitalized children. For this study, we evaluated data related to more than 55,000 weighted cases of AP in children admitted to U.S. hospitals. The HCUP databases have been used in a similar manner to achieve national level estimates of disease incidences and to investigate demographics, associated conditions, and outcomes of hospitalized patients within the United States [12]. We found an appreciable incidence of AP in hospitalized children during the most recent year available for this analysis and, importantly, an increasing incidence of this disease from 2000 to 2009. Several demographic characteristics of the group of children with AP were different from those children without this diagnosis. Excluding age, these differences were small and their clinical significance is probably not relevant.

The incidence of AP among hospitalized children in the United States during 2009 was 61.1 cases per 10,000 discharges. Considering only those cases with a primary diagnosis of AP, the incidence was 34.9. Overall, the highest incidences were in the oldest age group evaluated (16–20 years). Our estimated incidences are over ten-fold greater than those reported for pediatric cases of pancreatitis in previous studies. These differences can be attributed to different study goals and methodologies used to calculate the incidence of AP amongst the studies. Morinville and colleagues [5] investigated first time hospitalizations for AP as opposed to our study of any hospitalization. It is not possible for us to differentiate between first and subsequent episodes of acute pancreatitis occurring in individual patients due to the design of the HCUP-KID. Moreover, the authors of the previous study used their institution’s catchment pediatric population as the denominator to calculate the AP incidence, not pediatric hospitalizations as in our study [5]. An institution’s catchment population is greater than the population of hospitalized patients, thus an incidence based upon the former population will be significantly smaller than that based upon the later. Similarly, in calculating disease incidence, Nydegger and colleagues excluded recurrent episodes of AP and based the incidence on the general non-hospitalized pediatric population [6].

We found the incidence of AP to have increased between each of the four triennial periods of the study. This was observed for both the total number of weighted cases and the calculated incidence of AP (including primary and all-diagnoses). An increase in the number of cases of pediatric AP is consistent with earlier single-center studies [5], [6]. However, those studies reported data no more recently than 2004. We observed an increasing trend in the incidence of AP to the most recent year, 2009, available for analysis. Each age group included in this study has an increasing trend in the incidence of AP. We cannot directly address the reasons for the increase in AP cases given the constraints of this study, but we can speculate that the increasing trend is not due to a change in referral pattern or in the number of patients transferred from one institution to another as the HCUP-KID includes both urban and rural medical centers as well as teaching and non-teaching hospitals and less than four percent of AP discharges were transferred to another hospital (designated disposition within the HCUP-KID, data not shown). Though it is possible that physician awareness of AP increased during the period of our study, we postulate that a true increase in the incidence of AP in hospitalized children occurred during the period of this study due at least in part to an increase in one or several underlying etiologies. For example, the number of cases within the HCUP-KID of liver and biliary disease, the comorbid conditions we found to be the most highly associated with AP, increased by 60% from 2000 to 2009 (data not shown). Additionally, the incidence of IBD, another highly associated comorbid condition, has increased in children during recent years [13]–[15].

There was a significant decrease in the mortality and LOS for patients with AP for the period of our study. Improvements in these outcome measures suggest there has been an improvement in the care of patients with AP or of the underlying etiologies and associated comorbidities. Alternatively, it is possible that there has been an increase in hospitalizations of relatively mild cases of AP as a result of possible increases in awareness of and testing for the disease. The resultant proportional increase in mild cases would be expected to lead to decreases in mortality and LOS. During this same time period of improved mortality and LOS, hospital charges associated with AP increased. This increase may be reflective of the overall increase in medical charges in hospitalized patients within the United States. However, an increase in charges is not a universal occurrence observed for all common diseases in hospitalized children during a similar time period [12]. More aggressive (and expensive) care might have led to the improvement in mortality and LOS; this can only be determined with additional studies investigating the reasons for the increase in hospital charges.

Utilizing a cohort-matching protocol that incorporated both demographic and associated comorbid factors in the study design, AP was found to be an independent risk factor for lengthier hospital stays and greater charges. Thus AP is associated with a substantial disease burden in the hospitalized pediatric patient. However, AP was associated with a lower risk of mortality. The reason for the protective effect of AP on mortality is unclear. A possible explanation for this observation might be that the comorbidities associated with AP are primarily responsible for the observed mortality (e.g. trauma, sepsis) rather than AP itself.

In our investigation of the association of demographics and comorbid conditions with AP in hospitalized children we performed multivariable regression analyses to determine independent association of AP with these covariates. However, in our model we were unable to control for all possible factors associated with or etiologic for AP, such as medication usage. It is important to recognize that the associated comorbid conditions are not necessarily etiologic of AP, but can be the result of the disease itself (e.g. nutritional disorders, fluid and electrolyte imbalances). Therefore, these data reflect the clinical presentation and hospital course of children with AP. Liver and biliary diseases were the most highly associated comorbid diagnoses with AP. Children with liver and biliary diseases were 25 times more likely to also have a diagnosis of AP than children without liver and biliary diseases. This is consistent with previous, single-center experiences in children that identify these disorders as etiologies of AP [7]–[9], . Similarly, the other associated comorbid conditions identified in our study have been linked to AP [7]–[9],[16]–[19].

As discussed previously in this manuscript, there are several limitations to our study mostly due to the constraints of the database. The absence of longitudinal tracking of individual patients did not allow us to differentiate between first and repeat episodes of AP. The absence of pharmacy, laboratory and radiological data in the HCUP-KID resulted in the sole reliance on ICD9 codes to identify cases of AP while at the same time, an inability to comprehensively investigate etiologic agents of the disease. This also is the reason we were unable to assess the severity of AP through the use of severity scoring systems [21]; and we were unable to determine if children with AP required care in an intensive care setting.

## Conclusions

Our study demonstrates a recent increasing trend for AP in hospitalized children within the United States. The increase has occurred in all pediatric age groups over one year of age. The underlying reason for the increase in incidence of this disease is likely multifactorial and requires further exploration. Though the incidence of disease is increasing, there has been an improvement in the mortality and length of hospital stay of these patients. Despite these improvements, hospitalized children with AP have significant morbidity. These data provide a foundation for prospective multicenter studies designed to increase our understanding of the epidemiology of this disease in hospitalized children.
